# The effect of oral miltefosine in treatment of antimoniate resistant anthroponotic cutaneous leishmaniasis: An uncontrolled clinical trial

**DOI:** 10.1371/journal.pntd.0009241

**Published:** 2021-03-19

**Authors:** Masoumeh Tayyebi, Emadodin Darchini-Maragheh, Pouran Layegh, Bita Kiafar, Vahid Mashayekhi Goyonlo

**Affiliations:** Cutaneous Leishmaniasis Research Center, Mashhad University of Medical Sciences, Mashhad, Iran; University of Iowa, UNITED STATES

## Abstract

**Background:**

Recent circumstantial evidence suggests increasing number of Iranian patients with cutaneous leishmaniasis (CL) who are unresponsive to meglumine antimoniate (MA), the first line of treatment in Iran. Oral meltifosine was previously reported to be effective in visceral leishmaniasis as well CL. The current study is designed to determine efficacy and safety of oral miltefosine for the treatment of anthroponotic cutaneous leishmaniasis (ACL) cases who were refractory to MA in Iran.

**Methodology/Principal findings:**

Miltefosine was orally administered for 27 patients with MA resistant ACL with approved L.tropica infection, at a dosage of ∼2.5 mg/kg daily for 28 days. Patients were evaluated on day 14 and 28, as well as 3, 6 and 12 month post treatment follow up sessions. Laboratory data were performed and repeated at each visit. Data were analyzed using SPSS version 17. Twenty-seven patients including 16 men (59.25%) and 11 women (40.74%) with mean age of 28.56 ± 4.8 (range 3–54 years old) were enrolled. Total number of lesions were 42 (1–4 in each patient). Most of lesions were on face (76.19%). Mean lesions’ induration size was 2.38 ± 0.73 cm at the base-line which significantly decreased to1.31 ± 0.58 cm and 0.61 ±0.49 cm after 14 and 28 days of therapy, respectively (p value <0.05). At 12-months follow-up post treatment, 22 patients had definite/partial cure (81.48%) including 17 definitely cured patients, corresponding to a cure rate of 68% on per protocol analysis, and 62.96% according to intention to treat analysis. Recurrence of lesion was only occurred in one patient (3.70%). Nausea was the most subjective complication during the therapy (33.33%).

**Conclusion:**

Oral miltefosine could be an effective alternative for the treatment of MA-resistant ACL.

## Author summary

Leishmaniasis is endemic in 98 countries, mainly the Middle Eastern countries including Iran. More than 20 causative species of leishmania are known resulting in diverse disease manifestations, ranging from cutaneous localized ulcers to visceral leishmaniasis that can be fatal. Cutaneous leishmaniasis is usually a localized and self-limited disease, however, chronic non-healing lesions are one of challenging issues. Cutaneous leishmaniasis in Iran is mainly cause by two species: L. major-which causes anthroponotic cutaneous leishmaniasis (ACL)- and L.tropica-which causes zoonotic cutaneous leishmaniasis (ZCL)-. Conventionally, meglumine antimoniate (MA) is administered as standard treatment for ACL. However, resistant forms of ACL to MA are increasingly recognized in endemic areas. Antileishmanial drug miltefosine is capable of immunomodulation and evokes host immune responses. Therapeutic effects of oral miltefosine has been previously confirmed for treatment of visceral and muco-cutaneous leishmanisis. In the current study, oral miltefosine (∼2.5 mg/kg daily) was administered by 27 patients (42 lesions) with MA resistant ACL lesions. After 6-month follow-up, 68% of patients were definitely cured. Therefore, oral miltefosine could be an effective treatment choice for MA resistant ACL.

## Introduction

Leishmaniasis is a neglected tropical disease caused by different species of Leishmania [1]. Leishmaniasis is endemic in 98 countries, mainly developing ones. About 1.5–2 million new cases of leishmaniasis occur each year, of which 75% are cutaneous lesihmaniasis (CL) [1–2]. CL is endemic in Iran and the neighboring Middle Eastern countries. anthroponotic cutaneous leishmaniasis (ACL) and zoonotic cutaneous leishmaniasis (ZCL) caused by *L*. *tropica* and *L*. *major* respectively, are the most common types of CL in Iran [3].

Up to now, several topical and oral therapeutic approaches have been studied for treatment of CL. Most of these drugs have crucial disadvantages, including the length of treatment required, toxicity, painful injection, the emergence of resistance, nephrotoxicity, high cost and poor patient compliance [4]. Hence, the ambition to find an oral drug which could has effective formulation and short course of treatment without the prevalent limitations of toxicity and drug resistance remains unfulfilled.

Miltefosine—an alkylated phosphocholine- exerts its antimicrobial effects through affecting phospholipid membrane integrity and mitochondrial function of the microorganism [5]. Oral miltefosine has previously demonstrated a 95% cure rate in Indian visceral leishmaniasis [6–7].

In Iran, as endemic place for OWCL, therapeutic effects of oral miltefosine on ZCL have been studied previously. Mohebali and colleagues (2007) declared miltefosine at least as good as MA for the treatment of ZCL [8].

To date, no experimental study reported effects of oral miltefosine in treatment of ACL. Considering the variant therapeutic results in previous anti parasite reports, it is aimed to assess anti-leishmaniasis activity of systemic miltefosine in patients with ACL who were resistant to treatment with MA in Iran. The hypothesis was presumed as improvement in lesion induration size among MA resistant patients and complete/partial cure after the treatment period and during one-year follow-up.

## Material and methods

### Ethics statement

Written informed consent has been obtained from all the patients or their parents/guardian. Also, Female patients of childbearing age were included after giving written informed consent for effective contraception during therapy and until four months thereafter.

This study is approved by the institutional ethics board of Mashhad University of Medical Sciences, Mashhad, Iran. The protocol was also registered on Iranian Registry of Clinical Trials (IRCT20200302046673N1).

### Study design and population

It was an uncontrolled before-after study, conducted during 2018–2019 in two major leishmaniasis centers in eastern of Iran (Cutaneous Leishmaniasis Research Center and Ab-bargh PHC, Mashhad, Iran) on 27 patients with MA resistant ACL. ACL were already diagnosed according to clinical features and positive skin smear test. Moreover, L.tropica species was confirmed by Polymerase chain reaction (PCR) method in all cases. According to national committee of CL, MA resistant CL is defined as cases for whom two courses of MA treatment (at least one systemic course) have failed to cure CL lesions.

At first, patients who met the inclusion criteria were included in the study according to non-probability convenience sampling method. Those who had systemic underlying disease including hepatic, kidney or cardiovascular diseases, pregnant/breastfeeding mothers as well as dissatisfaction to continue were excluded from the study.

### Drug administration and follow-up

As pre-enrollment procedure, Sampling from lesion(s) and, if required, culture in Schneider and NNN media were carried out. Type, size, location and number of lesions were registered after positive parasitology and approve of *L*.*tropica*. Induration of each lesion was clinically evaluated by an experienced dermatologist and recorded in centimeter. Predesigned forms including demographic data and clinical history were filled for each patient.

Volunteers received miltefosine orally at a dosage of ∼2.5 mg/kg daily for 28 days as follows: <25Kg one capsule daily; 25–45 Kg two capsules per day; ˃45Kg three capsules per day. Patients were evaluated on day 14 and 28, as the last day of treatment, and lesions’ induration size as well as clinical adverse effects were recorded. Also, a complete blood count (CBC) and clinical chemistry (creatinine, aspartate aminotransferase (AST), alanine aminotransferase (ALT) were performed and repeated at each visit. Lesion induration was measured in length and width by ruler, and lesion area was calculated at enrollment and at each follow-up points during the trial. Lesions were also re-measured during follow-up visits 3 months, 6 months and 12 month post treatment. Clinical response as well as possible complications, recurrence rate and laboratory data were evaluated during follow ups. Standardized photographs were also to be taken from each lesion at different times of investigation.

Complete response (CR) for each lesion was defined as complete resolve of lesion induration and inflammation. All CR lesions were confirmed according to negative skin smear test. Due to systemic influence of the drug, “definite patient cure” required CR of all skin lesions in a patient. Patients who did not met definite cure criteria but had more than 50% improvement in all their lesions (according to induration size) were considered as “partial patient cure”.

Clinical failure was defined as less than 50% improvement of the lesion at least 3 months after therapy. Clinical relapse was an enlargement of a lesion or a new lesion in a previously definite cured patient.

### Data analysis

Quantitative variables are reported as mean ± SD. Qualitative variables are indicated as frequency and percentages. The kolmogorov-Smirnov test was used for evaluation of normality of the scores. Paired-samples t-test was used to compare the means within groups. X^2^ tests were used to determine whether there were any differences between members of patients, which were stratified according to healing. Statistical analysis was performed using SPSS version 17 software (Statistical Package for Social Science, version 17). For all analyses, P < 0.05 was considered statistically significant.

## Results

### Patients’ characteristics

The presenting characteristics as well as clinical data of the miltefosine recipients at the base-line are listed in Table 1.

Of the 27 patients, 16 patients (59.25%) were men. Mean age was 28.56 ± 4.8 (range 3–54 years old). Total number of lesions were 42 (1–4 in each patient). Most of lesions were on face (76.19%).

**Table 1:** Demographic data and clinical characteristics of 27 patients with acute anthroponotic cutaneous leishmaniasis registered in the study.

Characteristics	Values	Characteristics	Values
Gender Male Female	16 (59.25%) 11 (40.74%)	Lesion induration size (base-line) (Cm)	2.38±0.73
Age (years) Less than 10 10–20 20–30 More than 30	28.56±4.80 9 (33.33%) 2 (7.40%) 2 (7.40%) 14 (51.85%)	Lesion location Face Upper limbs Other site of body	32 (76.19%) 8 (19.04%) 2 (4.76%)
Previous systemic treatments Patients with 2 courses glucantime Patients with 3 courses glucantime	23 4	Number of lesions Patients with 1 lesion Patients with 2 lesions Patients with 3 lesion Patients with 4 lesions	42 17 7 1 2
Residence place Urban Rural	22 (81.48%) 5 (18.51%)	Lesion duration (years) More than 2 years 1–2 years Less than 1 year	3.45±0.76 19 (45.23%) 10 (23.80%) 13 (30.95%)

### Lesions induration size

**Fig 1:**
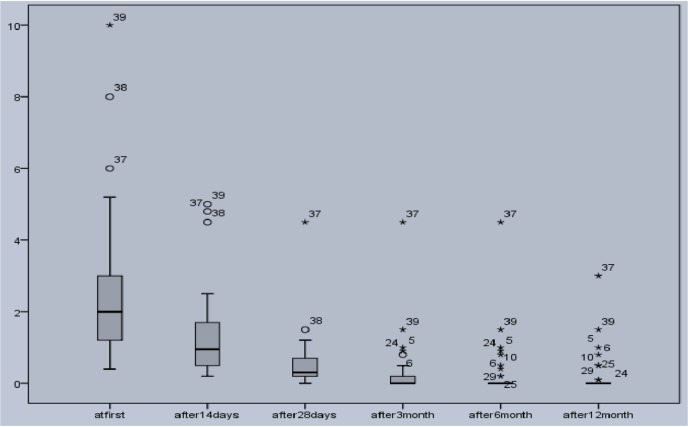
Box-plot graph of induration sizes in 42 cutaneous leishmaniasis lesions treated with oral miltefosine. X axis represents time of evaluation and Y axis represents lesion induration size (Cm).

Mean induration size was 2.38 ± 0.73 cm at the base-line of the study. Mean size after 14 and 28 days of therapy decreased to1.31 ± 0.58 cm and 0.61 ±0.49 cm, respectively which were significantly meaningful compared to the base-line sizes (p value <0.05 for both variables). Mean duration of lesions was 3.45±0.76 years (ranges between 8 months and 20 years). Lesion duration had no significant correlation with induration decrement after 12 months’ follow-up (p value: 0.64; r: 0.17). Fig 1, represents lesion size changes in evaluation times.

### Drug efficacy

Of 27 enrolled patients for systemic miltefosine treatment, 25 patients completed treatment. In the first follow up visit (three months after treatment) 23 patients (85.18%) gained complete or partial cure, including 14 definitely cured patients and 9 partially cured patients. Failure to therapy was recorded in two patients (7.40%). There were two dropouts (7.40%) due to creatinine level elevation, suspicious to drug induced nephrotoxicity in one patient and dissatisfaction to continue in one patient. In both second and third follow up sessions (sixth and twelfth month post treatment) 22 patients had complete or partial cure (81.48%) including 17 definitely cured patients, corresponding to a cure rate of 68% on a per protocol analysis, and 62.96% according to intention to treat analysis and 5 partially cured patients. Recurrence of lesions was only occurred in one patient (3.70%) during 6 month follow up. Table 2 demonstrates drug efficacy among the patients during the treatment and follow ups.

**Table 2:** Efficacy of oral miltefosine on 27 patients with acute anthroponotic cutaneous leishmaniasis.

Variable	Base line	Treatment		Follow up		
		14 days	28 days	3 month	6 month	12 month
**Lesion size (Cm)**	2.38 ± 0.73	1.31 ± 0.59	0.61 ± 0.23	0.26 ± 0.12	0.21 ± 0.08	0.03 ± 0.01
Definite patient cure^*^ (number)	-	0	0	14	17	17
Partial patient cure^**^/No cure^***^ (number)	-/-	9/18	19/8	9/2 (clinical failure)	5/2 (clinical failure)	5/2 (clinical failure)

*Patients with complete response in all the lesions according to induration and skin smear test.

**Patients with lesion(s) more than 50% improvement but not complete response.

***Patients with lesion(s) less than 50% improvement according to induration size.

### Adverse effects

Nausea, but not vomiting, was the most subjective complication during the study which detected in 9 (33.33%) patients. No other subjective complication was detected among the patients. Creatinine elevation was the only laboratory data complication which detected in one patient (3.70%) which raised from 0.80 mg/dl in the beginning to 2.0 mg/dl after 14 days. Patient with disturbed laboratory data were excluded from the study and referred to the nephrologist.

There were no significant differences in mean values of hematological, hepatic, and renal parameters in treatment groups.

## Discussion

About 75% of reported CL cases from Iran are zoonotic CL caused by L. major and the northeastern areas of Iran are meso-endemic for the disease [9].

The effect of systemic miltefosine on zoonotic CL have been previously discussed in literature. However, therapeutic effects of oral miltefosine on anthropontic leishmaniasis has not been reported yet.

The program to investigate miltefosine for new world cutaneous leishmaniasis (NWCL) was initiated in 2001 with an uncontrolled, open-label, dose-ranging study in Colombia, in which the per-protocol cure rate of 94% was reported by 2.5 mg/kg oral miltefosine per day after four weeks [10]. After that, Soto et al. (2004) reported significant more healing rate of miltefosine on NWCL compared with placebo [11]. In a randomized clinical trial conducted by Machado et al. (2010) 60 patients enrolled in miltefosine and 30 patients enrolled in pentavalent antimony group. After three month of administration, Oral miltefosine showed definite cure rate of 75% compared to 53.3% in antimony group. The incidence of adverse events was similar in both groups. The study demonstrated that oral miltefosine is more effective than standard pentavalent antimony and safe for the treatment of CL caused by Leishmania braziliensis in Brazil [12].

In a randomized clinical trial conducted by Mohebali and colleagues (2007), efficacy and safety of oral miltefosine were compared with MA in patients with zoonotic CL caused by L.major. Among 32 patients who received systemic miltefosine for one month, cure rate of 92.9% was reported on a per protocol analysis after three months. Cure rate of 83.3% was recorded for intramuscular MA on a per protocol basis. It was concluded that miltefosine could be apparently at least as good as MA in treatment of ZCL in Iran [8]. Even though the patients were not MA resistant in previous studies, the results are in accordance with our study. Oral miltefosine was also previously studied on new world CL among 72 Colombian patients with CL who had been treated at dosages of 50 mg/day for 20 days to 150 mg/day for 28 days, in which the 6-month cure rates were 81% on intention to treat basis and 94% on a per protocol basis with five patients being lost to follow-up [10]. Also, a placebo-controlled confirmatory study in 133 patients using a dosage of 150 mg/day for 28 days has been conducted in Colombia and Guatemala with remarkably similar results to cure rate obtained by miltefosine in other American CL studies [11].

Patients in the current study were refractory to conventional systemic therapy with MA which can explain the relative lower rate of definite patient cure compared to previous studies. It should be also noted that 69.04% of lesions had more than 1 year duration. Also, different efficacy rates may be related to the difference in cure rate between L. major versus L. tropica in Iran, as the former could be even self-limited and the latter causes more refractory lesions.

Since the number of cured patients did not differ at 6 and 12 month follow-ups, it seems that complete therapeutic effect of oral miltefosine could be observed 6 month post treatment. From this time on, it is possible to make a definite opinion on the patient’s response to oral miltefosine. However, follow up sessions are recommended due to check for recurrence.

Nausea but not vomiting was the most subjective complication during the therapy as recorded in 33.33% of patients. Averages of nausea (32.2%) and vomiting (21.5%) were previously observed in patients during two weeks after initiation of miltefosine treatment in a study conducted by Mohebali et al. (2007) [8]. Nausea and vomiting but not diarrhea is the main patients’ complaint specifically attributable to miltefosine. However, vomiting, diarrhea, anorexia, abdominal discomfort, headache, dizziness and somnolence have been reported in previous studies [13–14].

Despite significant creatinine elevation in one patient, significant differences were not recorded in any of mean laboratory values at the end of the therapy. Review of the adverse events associated with oral miltefosine mentions the adverse effects of mild-to-moderate aminotransferase and creatinine elevations as the most common possible laboratory disturbances [14]. Our results concerning the laboratory values of pre- and post-treatment of the patients show minor reversible liver enzymes elevations during treatment but they were not statistically significant. Thus, it could be clarified that in case of no obvious observed complication during oral miltefosine treatment, routine laboratory assessment is not mandatory.

Results of all Indian studies reported occasional elevations of transaminases, and creatinine returned to normal even though treatment continued [15]. Additionally, toxicological preclinical studies showed that there was no evidence of hepatotoxicity and renal failure in animal models. Therefore, it seems that the liver and kidney is not a major target organ for miltefosine toxicity [16].

Oral miltefosine regimen dose of ∼2.5 mg/kg/day for 28 days will be tolerated by majority of patients. Considering the early dropouts in the present trial, efficacy of miltefosine for definite patient cure in per protocol rate of 68% might be achieved. Total values of CR plus partial response among lesions could be estimated as 81.48%. Thus, longer treatment courses with systemic miltefosine might cause higher improvement rate among patients. The most important advantage of systemic miltefosine is its comfortable prescription. Nausea is common but episodes are usually short and tolerable, despite no prophylactic or symptomatic concomitant treatment is used.

The result of this study need to be seen in the sight of some limitations. A major limitation is lack of control group as well as low number of samples which restrict definite conclusion. The study was an open non-controlled case series. As all the included patients were considered as resistant to therapy, larger study group and suitable randomization were not possible. Longer treatment duration and follow-up sessions may also lead to more cured lesions and recommend for further studies.

## Conclusion

Our study documented that oral miltefosine is and effective and almost safe treatment choice for the treatment of MA-resistant lesions as well as chronic forms of ACL caused by L. tropica in Iran, based on parasitological and clinical follow-ups three, 6 and 12 months after treatment. Authors recommend additional large-scale controlled clinical trials to draw the conclusion.
